# Prevalence of ADHD Among Nonpsychotic Patients of Day-Care Centers and Comparison of Psychiatric Comorbidities Among Persons With and Without ADHD: A Pilot Study

**DOI:** 10.3390/jcm14041153

**Published:** 2025-02-11

**Authors:** Monika Szaniawska, Andrzej Kokoszka

**Affiliations:** 1 Poza Schematami, ul. Mickiewicza 6/8, 01-517 Warszawa, Poland; monika.szaniawska@gmail.com; 2 II Department of Psychiatry, Medical University of Warsaw, u. Kondratowicza 8, 03-242 Warszawa, Poland

**Keywords:** adult ADHD, neurodevelopmental disorder, comorbidity, inpatients

## Abstract

**Background/Objectives**: Adults with ADHD are more likely to develop other mental disorders. There are few data on the prevalence of ADHD among nonpsychotic patients treated with psychotherapy in day-care centers. This paper aims to assess persons with ADHD in this specific population and to compare psychiatric comorbidities in groups with and without ADHD. **Methods**: A total of 152 persons (men and women aged 18–58 years [M = 33; SD = 9.56]) were diagnosed according to the Mini International Neuropsychiatric Interview 5.0. (MINI). Personality disorders were assessed with SCID-II. ADHD was diagnosed according to a structured diagnostic history, DSM-IV-TR criteria, as well as CAARS and ASRS questionnaires. **Results**: A total of 47 persons (31%) met the criteria for ADHD in adults. Individuals with and those without ADHD did not differ in terms of education, employment, earnings, functioning on the labor market, satisfaction with their material status, being in a relationship, and divorce rate. Patients with ADHD more often had comorbidities such as major depression (34%) and personality disorders (31.9%), while for those without ADHD, anxiety disorders (58.1%) were predominant. The prevalence of personality disorders was higher in the group with ADHD and approached statistical significance (χ^2^ [1, N = 152] = 18.496; *p* = 0.06). Borderline and passive–aggressive personality disorders were also more frequent in this group. **Conclusions**: The prevalence of ADHD among patients treated in the day-care psychiatric center was much higher than in general population. In the group with ADHD, there was a higher prevalence of personality disorders, mainly borderline and passive–aggressive types. As only a small sample was analyzed in this single-center study, the presented findings need replication in similar settings.

## 1. Introduction

Attention-deficit/hyperactivity disorder (ADHD) is one of the most frequently diagnosed childhood disorders [1,2,3,4,5]. Nevertheless, its symptoms are often present in adulthood as well [6]. As early as in the late 1980s, studies demonstrated that symptoms of ADHD persist in about 70% of adolescents and 30–50% adults [7]. Numerous subsequent longitudinal studies have shown that in about two-thirds of individuals diagnosed with ADHD in childhood, the disorder persisted in its full-blown form through adolescence and adulthood [8,9,10,11,12]. Although many patients reported symptom reduction (primarily in hyperactivity), 60% of symptoms continued to cause inconvenience, and 90% of symptoms led to impaired functioning [13,14,15]. This genetically determined disorder seems to persist throughout the patient’s life, but it changes with age. This may be due to the fact that adults are more experienced and have learned how to deal with ADHD symptoms to a greater or less extent [13]. However, the symptoms may also be overlooked by psychiatrists and psychologists with no experience in diagnosing children and adolescents.

The prevalence of ADHD in adults across 20 countries has been recently estimated at 2.8%, ranging between 1.4% and 3.6% [16]. The first study on the prevalence of ADHD among adults was conducted in the United States by Barkley and colleagues on a sample of 720 persons who volunteered to renew their driver’s licenses. In that study, the prevalence of ADHD in adults was estimated at around 4.7% [12]. The National Comorbidity Survey Replication data from a study by Kessler et al. performed on more than 3000 respondents aged 18 to 44 indicated a 4.4% prevalence of ADHD in the United States [17]. In 2023, according to self-reported data from the National Center for Health Statistics Rapid Surveys System, 15.5 million adults in the United States (6%) had a current diagnosis of ADHD; approximately half of them were diagnosed at an age of ≥18 years [6]. Dutch epidemiological studies based on data from self-description questionnaires estimated the prevalence of ADHD at around 1% when the cut-off point for ADHD was set at six diagnostic criteria; however, with a cut-off point of four criteria, the prevalence was 2.5% [18]. Symptoms of ADHD were also significantly correlated with impaired functioning and other symptoms resulting from coexisting disorders.

ADHD is a life-long condition like diabetes, hypertension, bipolar affective disorder, or schizophrenia. Failure to initiate treatment due to lack of diagnosis and discontinuation of treatment may have multiple adverse consequences for the patient [19,20,21]. Adult patients with ADHD have a low chance to succeed in life as their ability to focus on the task or to behave according to social norms is usually insufficient [3].

So far, there have been no studies on the prevalence of ADHD among adults in Poland. Such studies were conducted only among children and adolescents and showed a prevalence similar to that reported in other European Union countries and the United States, i.e., ranging from 4.4% to 6.2% according to various data [22]. Based on that, we can expect that the prevalence of ADHD among Polish adults is similar to estimates from other European Union countries and the United States.

Undoubtedly, the diagnosis of ADHD automatically puts the patient at risk of developing other mental difficulties and disorders. Adults with ADHD are more likely to experience concomitant mental and somatic disorders [10]. On average, patients with ADHD have three concomitant disorders [23].

The most common comorbidities in adults with ADHD include the following:

1. Anxiety disorders (25–40%) [13,14,24,25,26].
2. Mood disorders (20–40%) [13,14,23,24,27,28].
3. Psychoactive substance and/or alcohol misuse (9–45%) [29,30].
4. Personality disorders (25% of children with ADHD develop a cluster B personality disorder in adulthood) [11,31].

When it comes to comorbidities, we can not only analyze the coexistence of other mental disorders in patients with ADHD but also assess how often ADHD is diagnosed in patients with other disorders. The risk of ADHD coexistence in individuals with at least three disorders is 8.3-fold higher than in the general population [28]. A few studies conducted so far indicate that, compared with the general population, adults with ADHD are more likely to be diagnosed with affective disorders, anxiety disorders, and addictions. Results of epidemiological and clinical studies suggest that ADHD affects about 10–20% of persons suffering from common mental problems [31]. Further studies show that this rate may be higher in certain clinical populations, such as patients reporting to forensic psychiatric centers and addiction and personality disorder clinics, which highlights the importance of ADHD screening in these high-risk populations [32].

In Poland, there are no special treatment centers for adults with ADHD. However, it does not mean that such patients do not exist there. Of note, ADHD symptoms do not disappear when a child reaches the age of 18 and becomes an adult under Polish law; about 30–50% of children do not grow out of their symptoms. Adult patients with ADHD usually receive a new diagnosis of a personality disorder, affective disorder, or anxiety disorder. Sometimes it is a misdiagnosis, and sometimes it is just to draw attention to disorders that coexist with ADHD. Because of their multiple professional, financial, and relational difficulties, these patients seek help from the public health system.

Group psychotherapy in day-care centers is the most accessible form of intensive psychological treatment within reimbursed psychiatric care in Poland. It means that adult patients with ADHD, due to their multiple comorbid disorders and emotional, psychological, occupational, and social maladjustment, probably most often go to centers providing treatment for personality disorders, affective and anxiety disorders, and addictions, i.e., psychiatric day-care centers. However, there are no Polish studies showing the estimated prevalence of ADHD in the Polish adult population.

In this article, we analyze baseline data from our pilot study (the effectiveness of psychotherapy in groups with and without ADHD will be discussed in a separate publication). This study aimed to

− estimate the prevalence of ADHD symptoms in adult patients referred to day-care psychiatric wards in Warsaw due to diagnoses other than ADHD;
− compare psychiatric comorbidities between groups with and without ADHD at baseline.

## 2. Materials and Methods

### 2.1. Participants

The most common form of psychotherapeutic treatment in a day-care setting is group psychotherapy. The treatment program usually includes a three-month stay, during which there are usually two one-and-a-half–hour group psychotherapy sessions per day for five days a week. In addition to psychotherapy, depending on the indications and severity of emotional problems, patients are concurrently managed pharmacologically by a psychiatrist working in the unit.

Therapy groups in the neurosis units participating in the study were led in a closed format. They usually consisted of 6 to 12 patients suffering from affective, anxiety, and/or personality disorders. The groups were usually heterogeneous. They were also peculiar in that the groups in the day wards are usually conducted in the morning hours (8 a.m. to 3 p.m.) and, therefore, prevent participants from engaging in professional work at the same time. The consequence of this is that working people are often unable to afford to be away from work for 3 months, and the ward receives people with highly severe problems that distort their professional life.

Therefore, the psychiatric day ward is most often visited by people whose condition is good enough not to require 24 h hospitalization but at the same time so severe that it prevents them from functioning socially, professionally, or in the family. Adults with ADHD function in a similar way, which is why the population of patients admitted to day psychiatric units is studied.

This study evaluated patients who took part in a three-month group therapy in the two selected day-care psychiatric wards in Warsaw (16 groups). Typically, such treatment took place Monday through Friday from 8 a.m. to 3 p.m. for 12 weeks. It was group treatment, and the groups ranged from 6 to 15 people. Sixteen groups were studied, and patients were mainly treated with dynamic, cognitive-behavioral, and eclectic therapy approaches.

Each patient met the following inclusion criteria:

- Admission to the ward based on a referral from a psychiatrist and an initial interview;
- Current diagnosis of Axis I anxiety disorder (based on the Mini International Neuropsychiatric Interview [MINI]);
- Diagnosis of a comorbid personality disorder (based on the Structured Clinical Interview for DSM-IV Axis II Personality Disorders [SCID-II]) or depressive disorder (based on MINI);
- Age between 18 and 65 years;
- Current health insurance.

Persons with organic lesions of the central nervous system and those currently dependent on psychoactive substances were excluded from the study.

Each patient was surveyed with a set of questionnaires at the beginning and at the end of therapy. Patients participated in three structured diagnostic interviews:

1. A diagnostic interview for ADHD.
2. A structured diagnostic interview for Axis II disorders, conducted on the basis of SCID-II.
3. The Mini International Neuropsychiatric Interview (MINI).

Course of the study:

The study began with the patient being informed of the purpose, duration, and consequences of the study. Patients were given written information about the study and requested to sign an informed consent form.

### 2.2. Materials

Structured diagnostic interviews developed for the specific purposes of this study and a set of standardized questionnaires were used:

- Diagnostic interview for ADHD: The diagnosis of ADHD in adults was primarily based on a thorough recollection of symptoms present in childhood and a proper evaluation of current symptoms and their impact on the patient’s life, based on the *Diagnostic and Statistical Manual of Mental Disorders*, Fourth Edition, Text Revision (DSM-IV-TR) criteria. For this purpose, a structured interview for ADHD in adults was developed on the basis of *Attention-Deficit Hyperactivity Disorder: A Clinical Workbook* (3rd Ed.) by Russell A. Barkley and Kevin R. Murphy [33].
- The Mini International Neuropsychiatric Interview (MINI) is a structured diagnostic tool designed to provide a rapid but accurate assessment of psychiatric disorders, particularly in the context of psychiatric diagnosis. MINI is a tool developed in the 1990s by Sheila M. Sheehan, Michael M. Lecrubier, and colleagues to diagnose psychiatric disorders according to DSM-IV criteria (previous version) and ICD-10 [34,35].
- Semi-structured interview for Axis II disorders, based on SCID-II: SCID-II allows for the assessment of twelve Axis II personality disorders classified in the DSM-IV and Appendix B to the DSM-IV. This interview was preceded by a self-description questionnaire filled in by the respondent, with 119 questions about the symptoms of personality disorders. Subsequently, the clinician interviewed the participant based on positively endorsed questions on the questionnaire [36].
- Conner’s Adult ADHD Rating Scales (CAARS)–Self-Report: Long Version. This is an adult questionnaire used to diagnose and assess the severity of ADHD symptoms in adults. The long version of the questionnaire, consisting of 66 questions, was used for the study [37].
- Adult ADHD Self-Report Scale (ASRS): A screening questionnaire for ADHD. It is a symptom inventory consisting of 18 questions based on the DSM-IVTR diagnostic criteria [17,38].

### 2.3. Statistical Analysis

Statistical analyses were performed using the SPSS Statistics 21.0 package. To compare differences between the groups of patients with and without ADHD, the χ^2^ test was used for nominal variables. A sample size calculator was used to assess the size of the group. The post-hoc power for this study and for the groups with and without ADHD was very high (post-hoc power, 99.5%).

## 3. Results

In total, 152 participants took part in the study: 97 women (63.4%) and 55 men (36.2%) aged from 18 to 58 years (M = 33; SD = 9.56). Patients read the description of the study and signed informed consent forms. Nobody refused participation.

The patients had the following primary diagnoses at admission: depressive episode and recurrent depressive disorder, 39 (25.7%); dysthymia, 4 (2.6%); manic episode, 2 (1.3%); mixed anxiety–depression disorder, 14 (9.2%); adjustment disorder, 21 (13.8%); panic anxiety disorder, 7 (4.6%); social phobia, 17 (11.2%); obsessive–compulsive disorder, 5 (3.3%); generalized anxiety disorder, 3 (2%); post-traumatic stress disorder, 5 (3.3%); somatoform disorder, 4 (2.6%); personality disorder, 31 (20.4%). A total of 47 participants (31%) met the diagnostic criteria for ADHD. Individuals with ADHD and those without ADHD did not significantly differ (χ^2^ test) in terms of education, employment, earnings, functioning on the labor market, satisfaction with their material status, being in a relationship, or divorce rate. The primary diagnoses of psychiatric comorbidities among persons with and without ADHD are presented in Table 1.

Detailed results are presented in Table 1.

In a semi-structured SCID-II interview, 125 (82.3%) of the study participants met the diagnostic criteria of at least one personality disorder, 21 (13.8%) had no personality disorder, and 6 (3.9%) could not be specifically diagnosed due to accidental destruction of the questionnaires, and only 146 persons are included in some more detailed analyses. Borderline personality disorder was diagnosed in 62 (40.8%) persons, avoidant personality disorder in 49 (32.2%), depressive personality disorder in 41 (27%), paranoid personality disorder in 38 (25%), obsessive–compulsive personality disorder in 35 (23%), narcissistic personality disorder in 33 (21.7%), passive–aggressive personality disorder in 29 (19.1%), dependent personality in 13 (8.6%), histrionic personality disorder in 8 (5.3%), schizotypal personality disorder in 7 (4.6%), anti-social personality disorder in 1 person (0.7%), and schizoid personality disorder also in 1 person (0.7%). The analysis of the diagnosed comorbidities in persons with ADHD revealed a higher risk of ADHD in patients diagnosed with personality disorders (χ^2^ [1, N = 152] = 18.496; *p* = 0.06). Cramér’s V was 0.349, which indicates a weak association.

The χ^2^ test showed a significant association between the frequency distribution of ADHD and diagnoses of personality disorders: χ^2^ (N = 152) = 7.501; *p* = 0.006. This means that persons with ADHD are more likely to have a personality disorder than those without ADHD. With a phi-coefficient of 0.227, the association was considered weak.

The results of the χ^2^ test also indicated a significant association between the distribution of ADHD and the diagnosis of borderline personality disorder: χ^2^ (1, N = 146) = 27.285; *p* ≤ 0.0001, which means that persons with ADHD had borderline personality disorder significantly more often than those without ADHD. The phi-coefficient of 0.432 was interpreted as a weak association between ADHD and diagnosis of borderline personality disorder. We also found a weak association between ADHD and passive–aggressive personality disorder: χ^2^ (1, N = 152) = 3.709; *p* = 0.054; phi-coefficient, 0.159.

The prevalence of personality disorders in groups with ADHD was also higher than in the group without ADHD, which was demonstrated by dividing the groups into clusters according to DSM-IV-TR; χ^2^ (6, N = 146) = 24.597; *p* ≤ 0.0001. Cramér’s V was 0.419, which was considered a weak association. Cluster B disorders coexisted with ADHD most frequently (diagnosed in 53% of individuals with ADHD); 18.2% of persons with ADHD had cluster A/cluster B/cluster C mixed personality disorders, and 15.9% also had cluster C disorders. However, in the group without ADHD, cluster C disorders were diagnosed in the biggest group of patients (29.4%). A total of 19.6% of patients without diagnosed ADHD had a cluster B diagnosis, and 19.6% of patients without ADHD had no personality disorders. The results are presented in Table 2.

## 4. Discussion

The European guidelines for the diagnosis and treatment of ADHD recommend that ADHD should be diagnosed and properly treated throughout patients’ lives [18,39,40,41]. Nevertheless, many professionals working in psychiatric wards are still unaware of the fact that ADHD often persists in adulthood, and they do not recognize clinical symptoms and life-long consequences of this disorder [42].

All 152 patients were referred to a day-care center for psychotherapy. No case of ADHD was recognized by the referring physicians. Of these, as many as 31% were diagnosed with ADHD in our study. This rate is much higher than the 2–5% estimated prevalence of ADHD in the general adult population. This means that individuals with diagnosed ADHD are overrepresented in the population of patients treated in psychiatric wards.

The results of our study seem to be consistent with the previous evidence for the coexistence of ADHD and other psychiatric disorders. A study of consecutive patients seen in an anxiety-disorders clinic found that 33% met criteria for adult attention-deficit/hyperactivity disorder (ADHD) [43]. A total of 27.9% of 129 consecutive patients referred to an anxiety disorder clinic were diagnosed with ADHD [44].

Another study on the prevalence of ADHD among patients receiving treatment for other psychiatric disorders was published by Pehlivanidis et al. in 2014. A group of 114 patients who turned for help to the outpatient clinic for anxiety and affective disorders were examined for the presence of ADHD. As a result, 19.3% of those patients were diagnosed with ADHD for the first time in their lives [45].

Several important conclusions can be drawn from our study. First of all, the coexistence of ADHD in persons receiving treatment for other chronic psychiatric disorders (anxiety disorders, affective disorders, personality disorders) is high (31% in our study and ranging from 19.3% to 33% in the cited literature). Second, those patients usually have undiagnosed and untreated ADHD, which certainly affects the ultimate effectiveness of the therapy. However, persons with ADHD who have not been diagnosed with and treated for ADHD in childhood rarely seek help with this in adult life. The disorder is also rarely diagnosed. Dutch studies showed that persons with ADHD seek psychiatric help for 12.5 years on average before they are properly diagnosed [13]. Third, the fact that ADHD is so prevalent among patients in whom standard outpatient treatment has proven to be insufficient and who are treated in the day-care psychotherapy ward due to excessive anxiety, depression, or relationship problems suggests that they should be screened for adult ADHD.

In this study, we investigated whether patients with ADHD differ from other patients of the day-care ward in terms of comorbidities. We found an association between the diagnosis on admission and the diagnosis of ADHD or lack of the diagnosis of ADHD. The strength of this association may be considered high. Patients with ADHD were most often admitted to the day-care ward with a diagnosis of depression (34%) and personality disorders (31.9%). Apart from that, ADHD was accompanied by the diagnosis of adaptive disorders (10.6%) and mixed depression–anxiety disorders (10.6%). Patients without ADHD were most often diagnosed with anxiety disorders (58.1%).

ADHD was most commonly diagnosed in patients with depression (53.8%), followed by social phobia (38.5%), generalized anxiety disorder (23.1%), and impulse control disorders (30.8%). Moreover, it was noted that the onset of the disorder occurred much earlier in persons with ADHD than in those without ADHD [44]. These data are particularly significant, as patient characteristics in that study were similar to those of our patients. In our study, the mean age of patients was 33 years (SD = 9.56); the majority of participants were female (64%) and were not in a relationship (55.9%). In the Canadian study, the mean age of patients was 33.1 years (SD = 12.5), the majority of participants were female (63.6%), and 49.5% of participants were not in a relationship [44]. Furthermore, there are studies [46] on patients turning to mental health clinics for depression that confirmed that depression was a significant comorbidity among adults with ADHD. Alpert examined 116 patients with ADHD at the beginning of their childhood and found that 16% of the study group met the criteria for ADHD in childhood and 12% met those criteria in adulthood.

The rate of coexisting depressive episodes and ADHD is very high in children, especially those who also have behavioral disorders [47]. In adults, it is estimated that 16–31% of those diagnosed with ADHD also meet the criteria for depression [23,48,49]; in Norwegian studies, 53% of adults with ADHD met the criteria for depression over the course of their lives, and 9% suffered from depression during the study [50]. Prospective longitudinal studies using a large sample of children demonstrated that 27% of patients diagnosed with ADHD in childhood had depression in adulthood [51] as well.

Due to such frequent co-occurrence of ADHD and depression, it is important to pay attention to the diagnosis of these two disorders. It seems necessary to answer two clinical questions:

1. Are the patient’s symptoms explained better with the diagnosis of ADHD or with the diagnosis of depression? (differential diagnosis)
2. Can we talk about the co-occurrence of the two disorders? (comorbidity)

The symptoms of adult ADHD and depression may overlap, especially when it comes to attention deficit, sleep disturbances, feeling tired, and lack of energy. The difference, however, is that these problems are part of the overall functioning of the individual in the case of ADHD, whereas in depression they are episodic and mainly related to periods of low mood. Thus, in ADHD, the symptoms persist from early childhood, and in depression we may capture their marked beginning. For example, it may be diagnostically important to ask the patient if they have had impaired functioning due to concentration disorders and difficulties in planning and completing tasks on time during periods of good and balanced moods.

The symptoms of ADHD that last for years contribute to numerous failures and losses. Persons with concentration issues, planning difficulties, who are impulsive, who do not predict the consequences of their actions, have less chance of success than their peers. Such a permanent experience of failure contributes to low self-esteem and little faith in the possibility to improve their situation. All of these are factors that lead to depression. Rucklidge and Kaplan presented several studies demonstrating that women with ADHD had significantly more symptoms of depression and anxiety, more often experienced stress, had lower self-esteem, and had a more external locus of control compared with women without ADHD [52]. Ten years later, it was found that all these characteristics also apply to men with ADHD. They had lower self-esteem and a more external locus of control and were more dissatisfied with their childhood than the control group and, surprisingly, than women with ADHD [24,43,53].

This study should be considered as a pilot one due to the following limitations. In fact, it is a single-center study of patients treated in two day-care centers that engaged staff from one university department, with a specific psychotherapeutic program and indirect inclusion criteria, as many patients look for programs focused on the specific methods of treatment. Day-care units are much more popular in Poland than in other countries, and it is the most available method of reimbursed psychotherapy. Our findings need verification in other day-care centers. The value of this study could be even greater if the occurrence of ADHD decreases the effectiveness of therapy. It would be strongly recommended to screen similar patients for ADHD before treatment initiation and probably to modify their therapy. Many patients had several comorbidities at admission. Due to a relatively small sample, the statistical analysis of comorbidities other than personality disorders among persons with and without ADHD was not justified.

## 5. Conclusions

The prevalence of ADHD among patients treated in two day-care psychiatric centers was much higher than in general population. In the group with ADHD, there was a higher prevalence of personality disorders, mainly borderline and passive–aggressive types. Due to a small sample analyzed in a single-center study, the presented findings need replication in similar settings to confirm data reliability.

## Figures and Tables

**Table 1:** Comparison of psychiatric comorbidities (primary diagnoses) in groups with and without ADHD.

Mental Health Disorder Other than ADHD	With ADHD	Without ADHD	Total
		Number of Patients (%)	
Depressive episode and recurrent depressive disorder	16 (34%)	23 (21.9%)	39 (25.7%)
Dysthymia	1 (2.1%)	3 (2.9%)	4 (2.6%)
Manic episode	0	2 1.9%)	2(1.3%)
Mixed anxiety–depression disorder	5 (10.6%)	9 (8.6%)	14 (9.2%)
Adjustment disorder	5 (10.6%)	16 (15.2%)	21 (13.8%)
Panic anxiety disorder	0	7 (6.7%)	7 (4.6%)
Social phobia	2 (4.3%)	15 (14.2%)	17 (11.2%)
Obsessive–compulsive disorder	0	5 (4.8%)	5 (3.3%)
Generalized anxiety disorder	0	3 (2.9%)	3 (2%)
Post-traumatic stress disorder	1 (2.1%)	4 (3.8%)	5 (3.3%)
Somatoform disorder	2 (4.3%)	2 (1.9%)	4 (2.6%)
Personality disorder ^1^	15 (32.%)	16 (15.2%)	31 (20.4%)
Total	47 (100%)	105 (100%)	152 (100%)

^1^ Diagnostic criteria of personality disorders according to SCID-II was met by 125 persons, but only in the case of 31 persons was it the primary diagnosis. In further analyses, all patients with the diagnosis of personality disorders diagnosis according to the SCID-II are included.

**Table 2:** Prevalence of personality disorders in patients with and without ADHD. Prevalence of personality disorders by clusters.

	Prevalence of Personality Disorders by Cluster							
	Cluster A	Cluster B	Cluster C	Cluster A and C	Cluster B and C	Cluster A, B, C	No Personality Disorder	Total
With ADHD diagnosis Number of patients (%)	1 (2.3%)	23 (52.3%)	7 (15.9%)	0	4 (9.1%)	8 (18.2%)	1 (2.3%)	44 (100%)
Without ADHD diagnosis Number of patients (%)	8 (7.8%)	20 (19.6%)	30 (29.4%)	1 (1%)	14 (13.7%)	9 (8.8%)	20 (19.6%)	102 (100%)
Total Number of patients (%)	9 (6.2%)	43 (29.5%)	37 (25.3%)	1 (0.7%)	18 (12.3%)	17 (11.6%)	21 (14.4%)	146 (100%)

Note: Diagnosis of ADHD and personality disorders: χ^2^ (6, N = 146) = 24.579; *p* ≤ 0.0001.

## Data Availability

The raw data supporting the conclusions of this article will be made available by the authors on request.
